# Effect of Foam Formulation on Magnesium Sulfate Cement Performance

**DOI:** 10.3390/ma19050913

**Published:** 2026-02-27

**Authors:** Dongqing Zhong, Hanxue Diao, Tao Pan, Guoliang Qu, Jianzhou Du, Jianyong Lu

**Affiliations:** 1 Institute of Straw Building Materials Ecology, Yancheng Institute of Technology, Yancheng 224002, China; 18852764235@163.com (H.D.);; 2 Jiangsu Sanling Abrasives Co., Ltd., Yancheng 224003, China; 3 Suzhou Loucheng New Materials Technology Co., Ltd., Suzhou 215443, China

**Keywords:** ultralight, modified magnesium sulfate cement, foam concrete, pore structure, thermal conductivity

## Abstract

In this study, the density of magnesium sulfate-based foam concrete was regulated by adjusting the foam dosage and the ratio of the foam stabilizer xanthan gum (XG) to the specialized foaming agent GX-7. The evolution of the pore structure was evaluated using bleeding rate tests and scanning electron microscopy (SEM). This investigation further elucidated the critical roles of density variation and pore morphology in determining the mechanical performance (compressive strength) and thermal insulation efficiency of the material. The results indicate that increasing the addition of high-stability modified foam significantly increased the pore density of the ultra-lightweight foam concrete while simultaneously reducing the average pore diameter. These microstructural changes led to a progressive decrease in bulk density, accompanied by a corresponding reduction in compressive strength. When the foam dosage reached 100% of the MgO mass, the material’s density decreased to 136.3 kg/m^3^, with a corresponding thermal conductivity of 0.081 W/(m·K). SEM micrographs revealed that, under these conditions, the pores exhibited a uniform morphology and well-defined structure, indicating an optimized pore architecture. However, when the foaming multiplier exceeded 125%, the frequency of bubble rupture increased markedly.

## 1. Introduction

Energy efficiency is central to the goals of green and low-carbon development, playing a vital role in reducing energy demand and mitigating greenhouse gas emissions. Globally, the building sector is a major contributor to energy consumption and carbon emissions. According to the International Energy Agency (IEA), buildings account for about 30% of global final energy consumption and 28% of energy-related CO_2_ emissions [1]. As Professor Pierrehumbert of Oxford University emphasized in 2019, “With regard to the climate crisis, yes, it’s time to panic” [2]. In this context, thermal insulation materials are fundamental components in the construction of ultra-low-energy buildings, as they significantly enhance energy efficiency and reduce operational carbon emissions.

Expanded polystyrene (EPS) and polyurethane foams are widely used organic thermal insulation materials for building envelopes. However, owing to their combustible nature, these materials often fail to meet stringent fire safety standards (e.g., Class A fire resistance), thereby posing significant fire hazards in practical applications [3]. In contrast, emerging high-performance inorganic insulation materials, such as aerogel and vacuum insulation panels, exhibit excellent fire resistance and ultra-low thermal conductivity. Nonetheless, their large-scale application is limited by factors including high cost, complex installation procedures, and concerns regarding long-term durability and performance stability [4,5,6]. Therefore, there is an urgent need to develop alternative insulation materials that are cost-effective, fire-safe, and environmentally friendly.

Lightweight cement-based composites represent a promising solution in this regard. Among them, magnesium-based cements have attracted increasing attention due to their low density, rapid setting, excellent fire resistance, and good compatibility with other materials [7]. In particular, magnesium oxysulfate cement (MOS) is synthesized from active MgO, MgSO_4_, water, and modifiers, with basic magnesium sulfate whiskers forming as the primary hydration products [8]. This cementitious system offers advantages such as light weight, inherent fire resistance, and a relatively low carbon footprint compared with ordinary Portland cement. Chau, C. K. and Li, Z. [9] conducted a systematic review, identifying the main hydration product of MOS cement as the 5Mg(OH)_2_·MgSO_4_·7H_2_O phase (5-1-7 phase). This phase serves as a lightweight material with a density of 600–1200 kg/m^3^ and a thermal conductivity of 0.15–0.35 W/(m·K).

Foam concrete is a mature, lightweight, and porous material formed by introducing stable foams into a cementitious base. In a study comparing foaming methods, Dvorkin, L. and Lushnikova, N. [10] found that the physical foaming method (using surfactants) demonstrated 25–30% higher foam stability than the chemical method (using H_2_O_2_). Furthermore, physical foaming achieved a porosity of 75–80% and allowed the dry density to be controlled within 350–900 kg/m^3^. Similarly, Newman, J. and Choo, B. S. [11] optimized the process parameters of precast physical foaming to improve the pore structure uniformity of the final MOS cement. Due to its porous and low-density structure, this material exhibits excellent thermal insulation [12,13,14,15], as the pore structure effectively hinders heat transfer [16,17]. It also possesses good fire resistance and sound insulation properties [18,19]. Extensive research has investigated foam concrete with various densities and binders. For example, Falliano et al. [20] produced foam concretes with dry densities ranging from approximately 350 kg/m^3^ to 850 kg/m^3^ using different cements and foaming agents. Akthar et al. [21] reported that ultra-lightweight foam concrete with compressive strength below 0.1 MPa typically exhibits porosity exceeding 90%. More recently, studies have combined MOS cement with foaming to develop lightweight insulation materials. Zhou et al. [22] reported MOS foam concrete with a dry density of 603 kg/m^3^ and a thermal conductivity of 0.14 W/(m·K). Similarly, Qin et al. [23] developed insulation panels incorporating rice husk and foam, achieving a dry density of 450.9 kg/m^3^ and a thermal conductivity of 0.1225 W/(m·K). Bouguerra [24] reported that foam concrete with a density of 300–1200 kg/m^3^ exhibits a thermal conductivity ranging from 0.075 to 0.35 W/(m·K). Ramamurthy et al. [12,25] proposed a density-based classification, where <600 kg/m^3^ is ultra-lightweight, 600–1200 kg/m^3^ is lightweight, and 1200–1800 kg/m^3^ is medium-weight. According to this classification, Jones & McCarthy [26] measured foam concrete with a density of 600 kg/m^3^ (classified as lightweight) to have a thermal conductivity as low as 0.14 W/(m·K)—significantly lower than that of traditional concrete (~1.8 W/(m·K)). They further emphasized that such lightweight properties can reduce foundation loads by 30–50%, leading to savings in materials and transportation costs.

From this, it can be seen that the dry density of the existing MOS foam concrete is usually above 300 kg/m^3^, and their thermal insulation performance and degree of ultra-lightweighting remain inferior to those of conventional organic insulation materials and advanced inorganic alternatives [26,27,28,29,30]. In particular, studies focusing on ultra-lightweight MOS foam concrete with densities below 200 kg/m^3^ are extremely limited, despite the fact that this density range is crucial for achieving exceptional thermal insulation performance.

Therefore, the novelty of this study lies in the use of modified magnesium oxysulfate cement as the binding matrix combined with a novel, highly stable modified protein-based foam employing a GX-7/XG composite foam stabilization system. This work systematically investigates the effects of foam characteristics and dosage on the physical, mechanical, and thermal properties of MOS foam concrete. The aim is to break through the conventional lower density limit of foam concrete (≥300 kg/m^3^) (JG/T 266-2011 [31]) and successfully fabricate ultra-lightweight MOS foam concrete with a dry density below 200 kg/m^3^. Furthermore, the mechanical performance, thermal insulation behavior, and pore structure characteristics of the resulting materials are comprehensively evaluated. By optimizing the foam stabilization system and regulating the macro- and micro-scale pore structure, this study aims not only to achieve ultra-lightweight materials with extremely low thermal conductivity but also to elucidate the underlying mechanisms by which foam stability and pore structure influence overall performance. This research seeks to fill the existing knowledge gap concerning MOS foam concrete in the ultra-lightweight density range and to provide a novel fire-resistant, lightweight, and high-efficiency insulation material for non-load-bearing building envelope applications, such as partition walls, roof insulation layers, and interior panels.

## 2. Materials and Methods

### 2.1. Raw Materials

Magnesium oxysulfate cement was used as the cementitious material for the preparation of foam concrete. Industrial-grade lightly calcined magnesium oxide (MgO, 120–180 mesh, activity: 65%, purity: 87.1%) and magnesium sulfate heptahydrate (MgSO_4_·7H_2_O, purity: 98.5%) were procured from China National Pharmaceutical Group Co., Ltd. (Beijing, China) Citric acid (CA) was supplied by Tianjin Zhonglian Chemical Reagent Co., Ltd. (Tianjin, China).

The high-stability modified composite foaming agent was prepared using deionized water and a magnesia-based foaming agent, GX-7, which was obtained from Shandong Magnesium Kart New Material Technology Co., Ltd. (Jinan, China) Xanthan gum (XG), used as a foam stabilizer, was a low-viscosity grade material purchased from Shandong Fufeng Food Co., Ltd. (Jinan, China).

### 2.2. Sample Preparation and Design

This study focused on the development of ultra-lightweight foam concrete based on modified magnesium oxysulfate (MOS) cement. To achieve ultra-low density and thermal conductivity, no aggregate was incorporated into the formulations. The processing method involved blending the cementitious matrix with preformed, highly stable foam. Systematic control of specimen density was achieved by varying parameters such as the type of foam stabilizer, its proportion, and its overall dosage while maintaining constant molar ratios of MgO to MgSO_4_ (O/S) and H_2_O to MgSO_4_ (W/S). The optimal foam formulation was initially selected based on its resistance to bleeding. Subsequently, a comprehensive evaluation was conducted to investigate the influence of foam content on key material properties, including compressive strength, thermal insulation performance, water absorption capacity, and macro- and microscale pore structure. The mix proportions of the lightweight magnesium sulfate foam concrete are summarized in Table 1. The magnesium oxysulfate cement formulation followed a fixed ratio of 2.25:1:1 (MgO:MgSO_4_:H_2_O), with citric acid added at 1% of the mass of magnesium oxide. The designations “F0, F25, F50, F75, F100, and F125” correspond to foam contents of 0%, 25%, 50%, 75%, 100%, and 125% relative to the mass of MgO, respectively. When the foam content exceeded 150%, the resulting ultra-lightweight magnesium oxysulfate foam concrete remained paste-like and failed to harden sufficiently for demolding. This surface collapse behavior is attributed to an insufficient volume of cement paste per unit area to adequately encapsulate the foam bubbles, thereby hindering their stabilization and preventing the formation of a coherent porous structure.

### 2.3. Test Method

The preparation of magnesium oxysulfate (MOS) foam concrete samples followed a multi-stage procedure. The main stages included (1) preparation of a highly stable modified foam, (2) preparation of a modified MOS cement paste matrix, and (3) mixing and molding of the foam concrete.

(1) Preparation of Modified Foam

A high-quality modified foam was prepared to ensure uniformity and stability in the final product. GX-7 was selected as the magnesium oxide-based foaming agent due to its rapid foaming rate, high efficiency, and ability to produce foam with uniform and stable bubbles. According to the formulations listed in Table 1, predetermined amounts of water, the foam stabilizer xanthan gum (XG), and the foaming agent GX-7 were accurately weighed. XG and GX-7 were sequentially added to the water and dispersed using a magnetic stirrer for 30 min to obtain a homogeneous and stable foam suspension.

(2) Preparation of Modified MOS Cement Paste

The solid matrix was prepared in the form of a modified MOS cement paste. The raw materials—MgO, MgSO_4_·7H_2_O, citric acid (CA), and water—were proportioned according to the mix designs presented in Table 2. First, MgSO_4_·7H_2_O and CA were dissolved in the measured amount of water under continuous stirring until a clear solution was obtained. Subsequently, MgO powder was gradually added to the solution and mixed thoroughly using a cement mortar mixer to form a homogeneous cement paste.

(3) Mixing and Molding

The prepared modified foam was then combined with the MOS cement paste. The two components were subjected to high-speed mixing to ensure complete homogenization and uniform distribution of the foam within the cementitious matrix, resulting in a fresh magnesium oxysulfate foam concrete mixture. Immediately after mixing, the fresh mixture was cast into steel molds with dimensions of 40 mm × 40 mm × 160 mm.

(4) Curing

**Figure 1:**
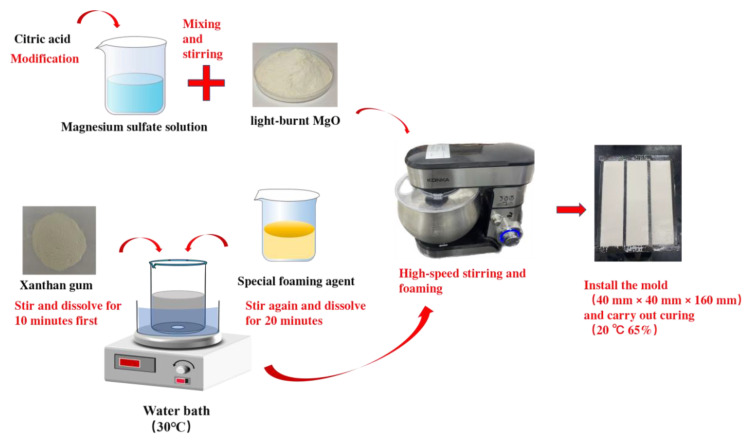
Diagram of the preparation process.

All specimens were cured under standard laboratory conditions (a temperature of 20 ± 2 °C and a relative humidity of 65 ± 5%) for designated ages of 7 and 28 days prior to testing for physical and mechanical properties. The overall fabrication procedure is schematically illustrated in Figure 1.

### 2.4. Test Methods

#### 2.4.1. Foam Stability Test

Bleeding water refers to the free water that exudes from a gas–liquid dispersion system—formed by mechanical stirring or physical foaming of an aqueous foaming-agent solution—due to gravitational drainage of the liquid film, inter-bubble drainage, and film rupture under standard temperature and humidity conditions within a specified standing time. It is an absolute quantitative indicator of the liquid-holding capacity of foam and is typically expressed as the volume of water released per unit volume of foam after complete collapse.

The bleeding rate is defined as the ratio of the bleeding water to the initial total water content of the foam system, expressed as a percentage (%). It serves as a relative standardized index for evaluating foam stability. The calculation formula is given as follows:

(1) $$\mathrm{bleeding}\mathrm{rate}=\frac{W_{\mathrm{fb}}}{W_{f\omega 0}}\times 100\%$$

where *W_fb_* is the actual bleeding water of the foam, expressed in milliliters (ml); *W_f__ω_*_0_ is the total mass of the liquid phase (foaming agent aqueous solution) used to prepare the foam, expressed in grams (g).

The foam bleeding rate was measured after standing times of 5 h and 9 h, in accordance with the test method specified in the Chinese National Standard JG/T 266-2011 [31].

#### 2.4.2. Dry Density

The dry density of the fabricated ultra-light magnesium oxysulfate foam concrete was determined in accordance with the Chinese standard JG/T 266-2011 [31]. The length, width, and height of each specimen were measured with an accuracy of 1 mm. For each dimension, two measurements were taken on opposite sides and one at the center, resulting in six measurements per specimen. The average of these measurements was adopted as the representative dimension. The specimen volume (V) was then calculated based on the averaged dimensions.

For drying, three specimens were placed in an oven maintained at 60 ± 5 °C until constant mass was achieved. Constant mass was defined as a mass variation of less than 1 g between two successive weighings conducted at an interval of 4 h. After drying, the specimens were transferred to a desiccator and allowed to cool to ambient temperature, after which the dry mass was recorded to the nearest 1 g.

The dry density was calculated using Formula (2):

(2) $$\rho_{0}=\frac{m_{0}}{V}\times 10^{6}$$

where *ρ*_0_ is the dry density (kg/m^3^), measured with a precision of 0.1 kg/m^3^; *m*_0_ is the dried mass of the specimen (g); and *V* is the specimen volume (mm^3^).

The dry density of each group was calculated as the arithmetic mean of the three measurements and reported rounded to the nearest 1 kg/m^3^.

#### 2.4.3. Flexural Strength

Flexural strength tests were conducted in accordance with the Chinese national standard GB/T 17671-1999 [32] using a WHY-300/10 microcomputer-controlled testing machine (Shanghai Hualong Testing Instrument Co., Ltd., Shanghai, China). The specimens were prepared as standard prisms with dimensions of 40 mm × 40 mm × 160 mm. Each specimen was properly positioned on the testing apparatus, and the loading head was adjusted to establish initial contact with the specimen surface before loading. The test was performed under control at a constant loading rate of 50 ± 5 N/s until failure. The maximum load at failure was recorded, and the average value obtained from three replicate tests was reported as the flexural strength for the corresponding curing age.

#### 2.4.4. Compressive Strength

Compressive strength tests were performed in accordance with the Chinese industrial standard JG/T 266-2011 (Foam Concrete) [31] using the same WHY-300/10 microcomputer-controlled testing machine. Prismatic specimens (40 mm × 40 mm × 160 mm) were centrally positioned on the lower platen of the testing machine. The upper platen was carefully adjusted to ensure uniform contact with the specimen surface prior to loading. A constant loading rate of 2400 ± 200 N/s was applied until failure. The maximum load was recorded for each specimen, and the average of three measurements was reported as the compressive strength at the designated curing age.

#### 2.4.5. Water Absorption Rate

The water absorption test was conducted in accordance with JG/T 266-2011 [31]. Three prismatic specimens (40 mm × 40 mm × 160 mm) were dried in an electric forced-air oven at 65 ± 5 °C until constant mass was achieved, defined as a mass variation of less than 1 g between two successive weighings at 4 h intervals.

After cooling to room temperature, the specimens were immersed in water at 20 ± 5 °C following a staged saturation procedure: immersion to one-third of the specimen height for 24 h, followed by two-thirds immersion for another 24 h, and finally full immersion with a water level at least 30 mm above the specimen surface for an additional 24 h. After removal, surface water was wiped off using a damp cloth, and the specimens were immediately weighed to the nearest 1 g.

The water absorption rate was calculated using Formula (3):

(3) $$W_{R}=\frac{m_{g}-m_{0}}{m_{0}}\times 100\%$$

where *W_R_* is the water absorption rate, accurate to 0.1%; *m*_0_ is the dry mass (g); and *m_g_* is the saturated mass (g).

**Figure 2:**
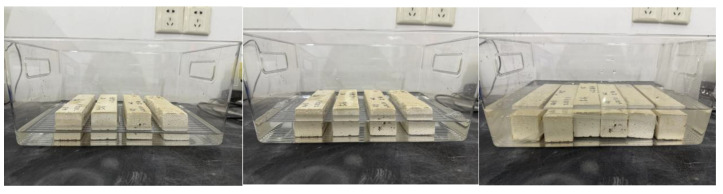
Partially submerged test blocks.

The reported water absorption value represents the arithmetic mean of three specimens. Due to the low density of certain specimens, flotation occurred during immersion; therefore, nonwoven fabric and additional counterweights were used to ensure complete submersion and saturation throughout the test period. As shown in Figure 2.

#### 2.4.6. Thermal Conductivity

Thermal conductivity was measured in accordance with the Chinese National Standard GB/T 32064-2015 [33] (“Test Method for Thermal Conductivity and Thermal Diffusivity of Building Materials with Transient Plane Source”). Specimens with dimensions of 300 mm × 300 mm × 30 mm were prepared. After standard curing for 28 days, the specimens were dried at 40 ± 5 °C to constant mass and subsequently conditioned in a controlled environment (296 ± 1 K, 50 ± 10% relative humidity) until hygroscopic equilibrium was achieved. Thermal conductivity was then measured using a transient plane source thermal conductivity analyzer.

#### 2.4.7. Microstructure

The pore microstructure was examined using a field-emission scanning electron microscope (Nova Nano SEM 450, FEI Company, Hillsboro, OR, USA). Representative specimens were fractured into small fragments and dried at 40 °C for 48 h. The dried fragments were stored in vacuum-sealed plastic bags until analysis. Prior to SEM observation, all samples were sputter-coated with a thin gold layer to improve electrical conductivity and image quality.

## 3. Results and Discussion

### 3.1. Bleeding Rate Test

Under the coupled effects of gravity and buoyancy, freshly generated foam undergoes coalescence and coarsening, which promote gas diffusion and ultimately lead to collapse of the foam structure. Therefore, lower foam drainage corresponds to higher foam stability. According to the Chinese industrial standard JG/T 266-2011 [31], “1-h bleeding water” refers to the volume of liquid separated from the foam after standing for 1 h, while “1-h settlement distance” denotes the downward displacement of a reference marker placed inside the container over the same period. Both parameters are widely used as quantitative indicators for evaluating the stabilizing effects of foam stabilizers.

**Figure 3:**
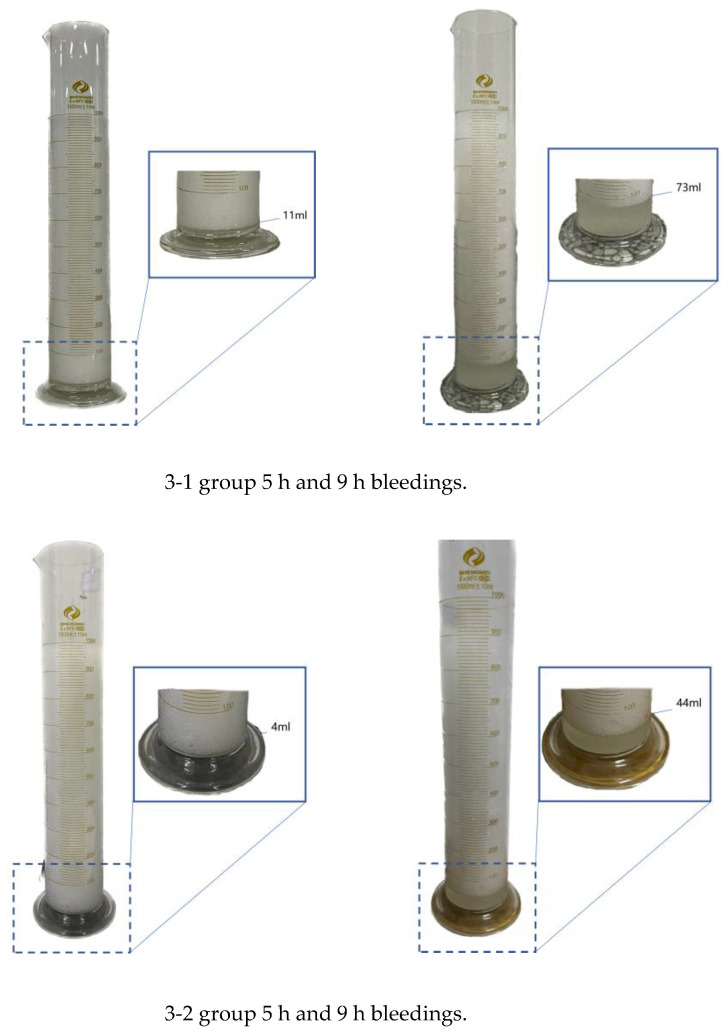

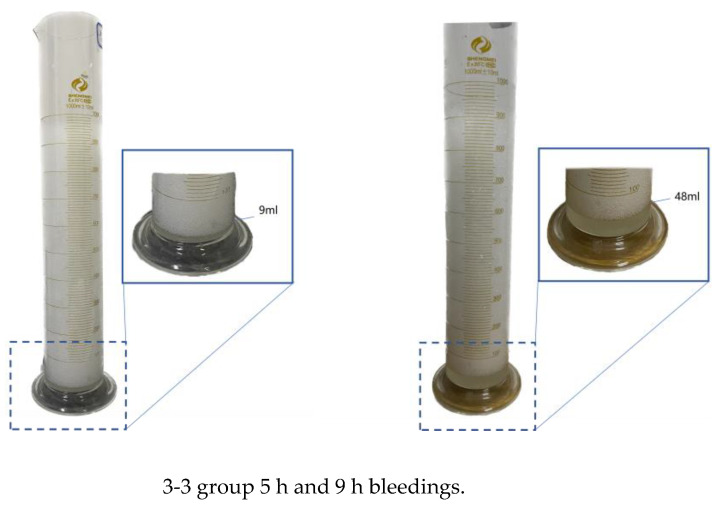
Group 5 h and 9 h bleedings.

Considering that the stabilizing performance of foam stabilizers may vary with foam formulations, the water retention capacity of the ultra-lightweight foam system was systematically evaluated. For each mixture, three cylindrical containers filled with freshly prepared foam were placed in a quiescent environment, and the volume of expelled liquid was measured after 5 h and 9 h. Representative results are shown in Figure 3.

**Figure 4:**
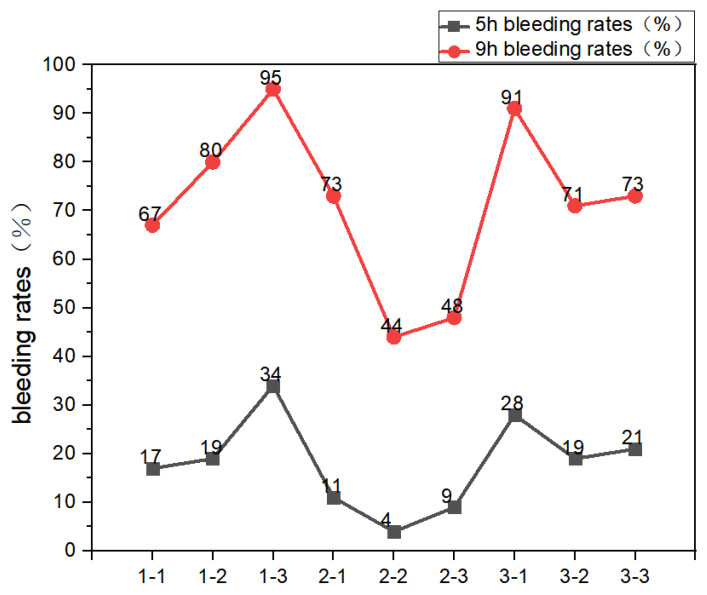
5 h and 9 h bleeding rates.

The data presented in Figure 4 and Table 3 indicate that the foam exhibits the highest stability in the fresh state when the foaming agent and foam stabilizer are used at a mass ratio of 1:1. An excessive dosage of the foaming agent produces bubble volumes that exceed the encapsulation capacity of the cementitious matrix, thereby promoting bubble coalescence, rupture, and loss of structural continuity. This results in over-foaming, increased porosity, reduced stability, pronounced liquid drainage, and severe bleeding and stratification of the slurry, ultimately impairing casting uniformity. Conversely, an insufficient foaming-agent dosage limits bubble generation due to inadequate foaming capacity, restricting density reduction and preventing the formation of an optimized pore structure. Such mixtures exhibit low porosity, higher density, and poor workability owing to excessive paste viscosity.

Similarly, an excessively high proportion of the foam stabilizer markedly increases slurry viscosity, hindering uniform bubble dispersion, delaying hydration, and complicating mixing and casting operations. Elevated viscosity also obstructs the release of entrapped air, leading to localized stress concentrations and shrinkage cracking. In contrast, an insufficient amount of stabilizer weakens the foam liquid film, rendering it vulnerable to rupture under shear stresses imposed by the cement paste or external disturbances. This accelerates bubble coalescence and results in heterogeneous pore structures, macrovoid formation, mold collapse, and a non-uniform density distribution, ultimately causing large variability in compressive strength.

### 3.2. Dry Density

**Figure 5:**
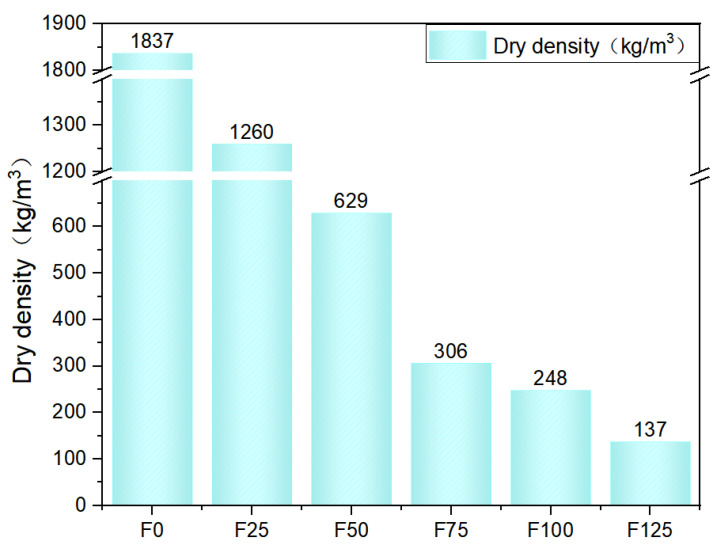
Dry density.

The dry densities measured for each mixture series are summarized in Table 4 and graphically presented in Figure 5 for comparison. As shown in the figure, increasing the foaming ratio initially results in a pronounced reduction in dry density, followed by a gradual plateau. This trend can be attributed to the introduction of a larger volume of air into the mixture at higher foaming ratios, which effectively dilutes the solid skeleton of the material. More specifically, an increased foaming ratio corresponds to a higher volume fraction of low-density air (i.e., increased porosity) and a reduced proportion of the high-density solid matrix, thereby leading to a decrease in the overall bulk density.

### 3.3. Flexural Strength

Flexural strength tests were conducted in accordance with the UNI EN 196-1 standard [34] using a three-point bending configuration with a span length of 100 mm. Loading was applied under force control at a constant rate of 50 N/s. Specimens from each mixture were initially cured for 7 days at 20 ± 5 °C and subsequently conditioned for up to 28 days at a relative humidity of 60 ± 5%. For each curing age, three prismatic specimens were tested. During loading, the applied force was oriented perpendicular to the casting surface of the concrete.

**Figure 6:**
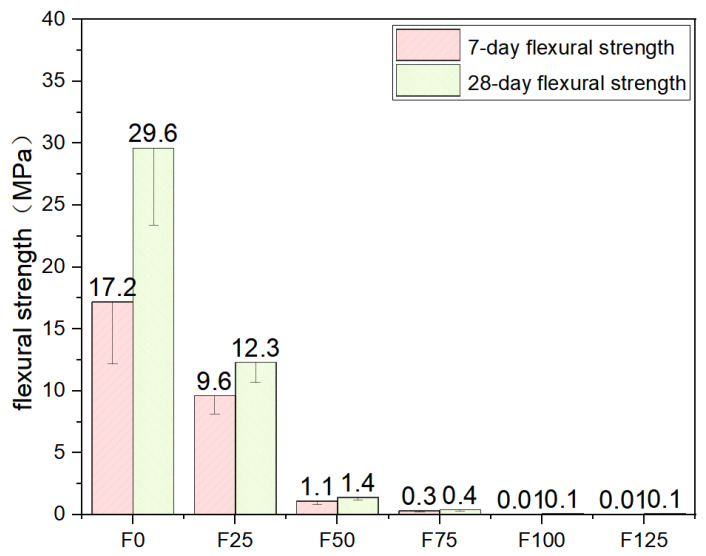
Flexural strength.

As shown in Figure 6, The flexural strength of composite materials with different mix formulations (F0 to F125) was systematically evaluated after 7 days and 28 days of curing, and the results are presented in the corresponding figure. At 7 days, the flexural strength ranged from 0.01 MPa for F100 and F125 to 17.2 MPa for F0. After 28 days of curing, most formulations exhibited notable strength development, with flexural strength values increasing to between 0.1 MPa (F100 and F125) to 29.6 MPa (F0). Notably, F0 consistently exhibited the highest mechanical performance at both curing ages, achieving 17.2 MPa and 29.6 MPa at 7 and 28 days. Moderate foaming levels (e.g., F25 and F50) retained relatively comparable flexural strength; however, a substantial decline in strength was observed as the foaming ratio increased. Beyond F50, strength development was significantly constrained, which is likely attributable to insufficient matrix continuity and weakened interfacial bonding within the highly porous structure. These results indicate that further modifications—such as matrix reinforcement or interfacial enhancement—are required to improve the flexural performance of high-porosity foam cement composites, thereby achieving a more effective balance between sustainability objectives and structural performance requirements.

### 3.4. Compressive Strength

Following the flexural tests, the specimens were subjected to compressive strength evaluation, with six specimens tested for each mixture proportion. Compressive tests were conducted in accordance with the UNI EN 196-1 standard under force-controlled loading. However, the loading rate was reduced to 50 N/s and maintained constant throughout the test to accommodate the anticipated low compressive strength of the highly porous material, thereby ensuring reliable measurements within its specific mechanical response range [33]. All compression tests were performed using a Zwick-Line mechanical testing system equipped with a 1 kN load cell.

**Figure 7:**
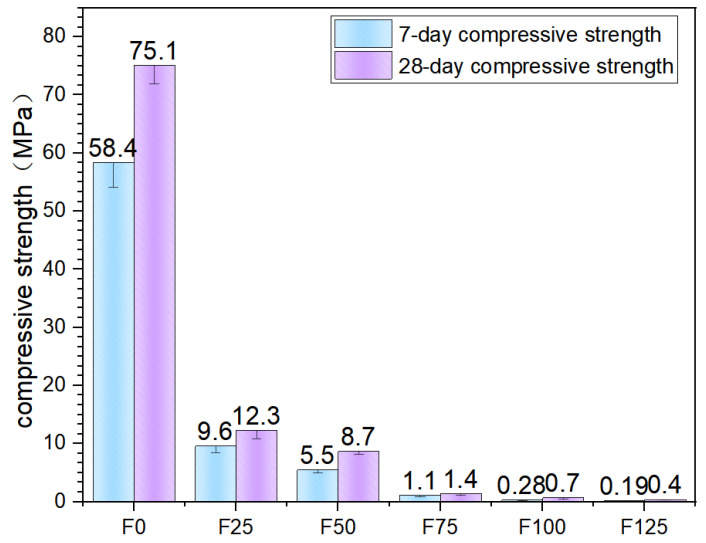
Compressive strength.

In compliance with UNI EN 196-1, the specimens were positioned such that the applied load was perpendicular to the casting direction. The progression of the compression tests for each mixture group is illustrated in the corresponding figures. The results obtained from six representative specimens, selected according to the foam content gradient, are highlighted in Figure 7. Individual test results are presented together with the average compressive strength values and the coefficients of variation calculated for each mixture series.

The results indicate a progressive decline in compressive strength with increasing foam content. Specifically, compressive strength decreases sharply at lower foam contents and then exhibits a progressively moderated rate of reduction. This behavior can be attributed to two primary mechanisms. First, at foam contents below 100%, the increase in porosity within the modified magnesium oxysulfate matrix significantly reduces its load-bearing capacity. In contrast, when the foam content increases from 100% to 150%, the formation of additional pores becomes limited, resulting in only a marginal further decrease in the compressive strength of the ultra-lightweight magnesium oxysulfate foam concrete [35]. Second, increasing foam content leads to an expansion of the overall matrix volume, thereby reducing the effective content of modified magnesium oxysulfate cement per unit volume. The reduced cement content is insufficient to fully encapsulate the foam surfaces and to form a continuous and stable skeletal framework. Moreover, the formation of the primary strength-contributing hydration product—the 5·1·7 phase (5Mg(OH)_2_·MgSO_4_·7H_2_O)—is significantly suppressed due to cement dilution. The combined effects of matrix expansion and reduced hydration product formation substantially compromise the compressive resistance of the material.

In summary, the compressive strength of ultra-lightweight magnesium oxysulfate foam concrete decreases markedly at lower foam contents and subsequently tends to stabilize as foam content further increases. These findings indicate that additional measures must be adopted in the later stages of pure foam cement system design to enhance the compressive strength of the matrix, thereby achieving an appropriate balance between sustainability objectives and mechanical performance requirements.

### 3.5. Water Absorption

**Figure 8:**
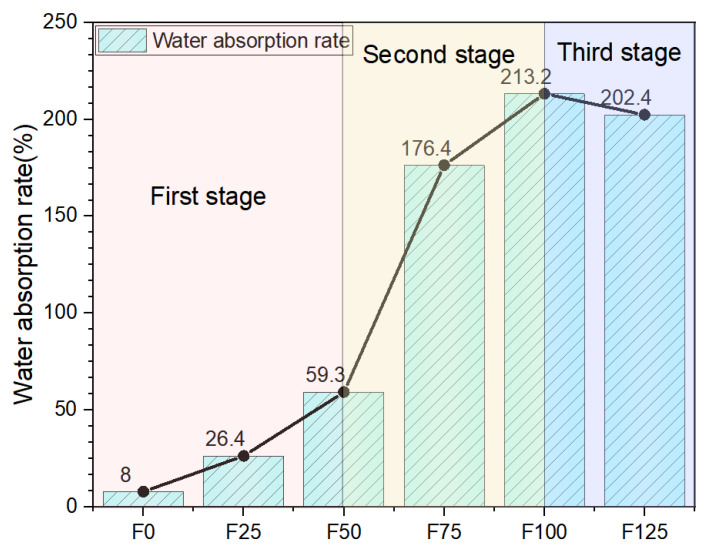
Water absorption rate.

As shown in Figure 8, the water absorption of the specimens exhibited three distinct stages with increasing foaming ratios. In the range from F0 to F50, the water absorption increased steadily and gradually. This stage corresponds to the initial phase of foaming, during which the pore content remained relatively low. Between F50 and F100, the water absorption increased rapidly. At this point, the foaming process neared completion, resulting in a more integrated and uniform pore network, while the overall density of the material decreased significantly. From F100 to F125, the foaming ratio became excessive, leading to bubble coalescence and rupture, which caused the water absorption rate to decline.

The foaming agent content was inversely correlated with the specimen density. Higher dosages of the foaming agent led to a significant increase in mass-based water absorption and a pronounced decrease in both flexural and compressive strengths, particularly under wet conditions. Notably, magnesia-based materials inherently exhibit poor water resistance. After one month of water immersion, all specimens softened and lost virtually all their mechanical strength. This degradation was attributed to the formation of numerous uniform, fine, and largely impermeable pores upon the addition of foam to the magnesia-based slurry. Owing to the intrinsically low water resistance of the matrix, the material readily absorbs water, which accumulates within these closed pores as isolated reservoirs. Because water ingress is a continuous process, the moisture content gradually increases until saturation is reached.

Furthermore, prolonged contact of the pore walls with stored water, combined with the poor durability of the hydrated phases in aqueous environments, accelerates microstructural deterioration. This results in a rapid decline in the mechanical properties and, in severe cases, complete disintegration of the material.

### 3.6. Thermal Conductivity

**Figure 9:**
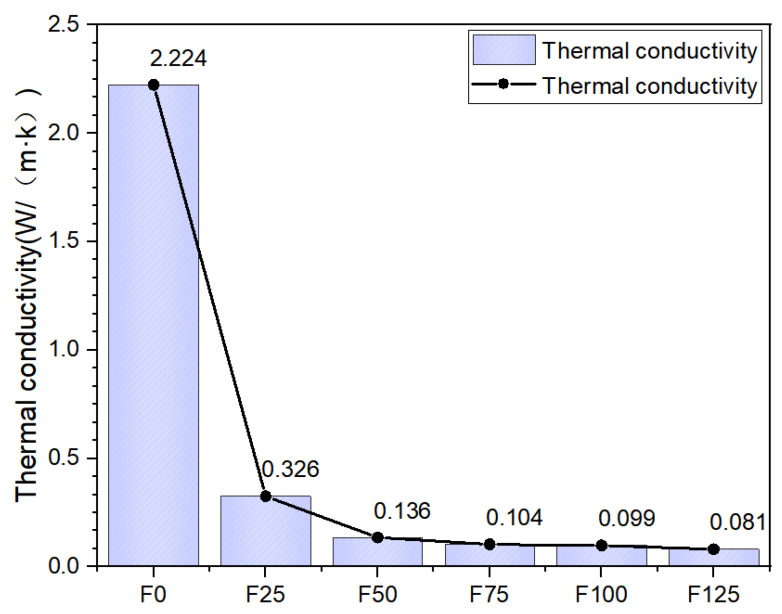
Thermal conductivity.

Magnesia-based foam slurries with varying foaming agent contents were cast into test panels measuring 300 mm× 300 mm × 30 mm. Following standard curing for the designated periods, the specimens were dried at 60 ± 5 °C until a constant mass was achieved. The thermal conductivity was measured using a ZDR-3030/6060 thermal conductivity tester from Liaoning Hexing Zongheng Mechanical and Electrical Equipment Co., Ltd. (Shenyang, China) in compliance with the Chinese standard GB/T 10294-2008 [36]. The corresponding test results are presented in Figure 9.

As shown in Figure 9, the bulk density of the specimens decreased with increasing foaming agent content, which was accompanied by a corresponding reduction in thermal conductivity. This observation demonstrates that, under given temperature conditions, the thermal conductivity of the material is predominantly governed by its bulk density and microstructural features, specifically pore size distribution, pore uniformity, pore density, and pore connectivity. A lower apparent density, combined with a reduced fraction of interconnected pores, effectively restricts heat transfer pathways and thereby enhances the thermal insulation performance of the material.

In building insulation systems, thermal conductivity is a critical parameter for evaluating the thermal insulation performance of materials—lower thermal conductivity indicates superior insulating capability. The relationship between foam content and thermal conductivity in ultra-lightweight magnesium oxysulfate (MOS) foam concrete is graphically presented. As shown, higher foam content leads to a progressive decrease in thermal conductivity. Specimens without foam (Group F0) exhibited a thermal conductivity of 2.224 W/(m·K), while the thermal conductivity of Group F25 decreased to 0.326 W/(m·K), representing an 85.34% reduction. When the foam content was increased to 100% (Group F100), the thermal conductivity reached 0.099 W/(m·K), corresponding to a 95.55% decrease relative to F0. A further increase to 125% foam content (Group F125) resulted in a thermal conductivity of 0.081 W/(m·K), which is 96.36% lower than that of F0.

This substantial reduction in thermal conductivity can be attributed to the incorporation of a high volume of air-filled pores within the magnesium oxysulfate cement matrix as the foam content increases. These pores predominantly form closed-cell structures, which significantly increase the number of voids and effectively impede heat transfer through the material, thereby markedly lowering its thermal conductivity. The results demonstrate that increasing the foam content effectively reduces the thermal conductivity of ultra-lightweight MOS foam concrete until it eventually approaches a limiting value, beyond which further enhancement becomes marginal.

Table 5 compares the thermal conductivity of the insulation materials developed in this study with that of conventional construction insulation materials. As shown, the performance of the materials produced here compares favorably with commercial products and meets practical requirements for insulation. Furthermore, in contrast to many conventional materials which may raise concerns regarding environmental impact and human health, the materials developed in this work offer a more sustainable and potentially safer alternative, justifying their further investigation. Furthermore, based on this foundation, the subsequent enhancement of the mechanical properties of this material is the key focus.

### 3.7. Characterization of Micropore Structure

**Figure 10:**
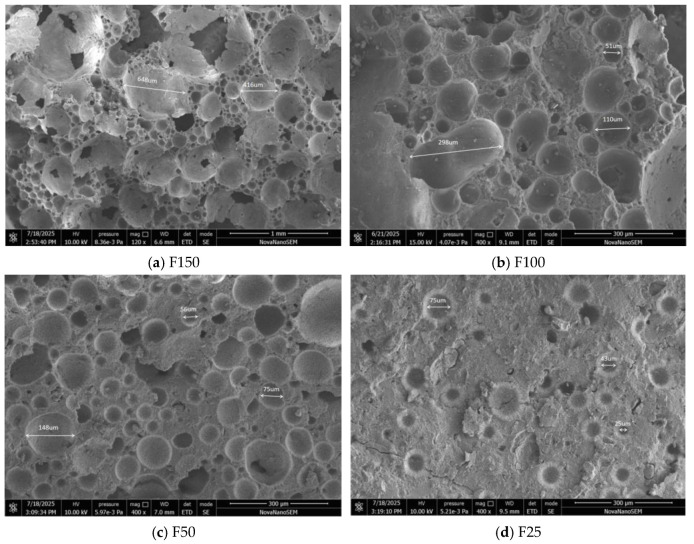
Micrographs of pore structure at each expansion ratio.

To elucidate the effect of the foaming multiplier on the microstructure, samples with different foaming multipliers were examined using scanning electron microscopy (SEM), and the average pore size was statistically analyzed via image analysis. The key findings, as shown in Figure 10 and Table 6, demonstrate that as the foaming multiplier increases, the average pore size initially increases and then stabilizes, accompanied by a significant broadening of the pore size distribution. These microstructural changes are intrinsically linked to the evolution of macroscopic properties.

#### 3.7.1. Trend in Pore Size Variation

As the foaming multiplier increased from 0% (reference sample F0) to 100%, the introduction and expansion of air bubbles led to a significant increase in the average pore size from less than 50 μm (F0, primarily composed of microscopic gel pores) to approximately 280 μm (F100). This growth positively correlates with the foam content, as a greater volume of gas introduced into the paste coalesces to form larger pores. However, when the foaming multiplier was further increased to 150%, the average pore size showed only a modest increase to about 320 μm, indicating a markedly reduced growth rate. This suggests that the paste’s capacity to encapsulate foam approaches saturation at high foaming multipliers. Excess foam is more likely to cause coalescence, rupture, or escape of adjacent bubbles rather than consistently forming larger, discrete pores.

#### 3.7.2. Pore Size Distribution and Pore Structure Homogeneity

The uniformity of the pore size distribution is a critical factor influencing material performance. Samples with low foaming multipliers (25%, 50%) exhibited relatively narrow pore size distributions, indicating well-dispersed foam within the paste and the formation of a dense porous network. In contrast, samples with foaming multipliers of 100% and 150% showed significantly broadened distribution curves, featuring a substantial population of pores larger than 500 μm alongside a minority of pores smaller than 100 μm. This indicates a deterioration in pore structure homogeneity, primarily attributable to pore coalescence and structural instability induced by excessive foam.

#### 3.7.3. Correlation Between Microstructure and Macroscopic Properties

The increase in average pore size and the broadening of the distribution directly impact the macroscopic properties. Primarily, the increase in average pore size and the presence of exceptionally large pores (>500 μm) significantly compromise the continuity of the solid skeleton, creating stress concentration points. This is the primary reason for the sharp decline in compressive and flexural strength with an increasing foaming multiplier. Secondly, air pores, particularly closed pores within an average size range of 100–400 μm, act as effective thermal insulation units. The moderate increase in pore size (up to 100%) effectively reduced the thermal conductivity. However, for the 150% foaming multiplier, although the average pore size continued to increase, the reduction in thermal conductivity was less pronounced. This is attributed to deteriorated pore homogeneity and increased open porosity due to pore wall rupture. Finally, increased pore size and enhanced pore interconnectivity provide more pathways for water ingress, leading to a significant increase in water absorption for samples with high foaming multipliers (F100, F150).

In summary, the foaming multiplier primarily governs the microstructure of magnesium oxysulfate foam concrete by controlling the average pore size and pore size distribution. Within the range of 0% to 100%, the system effectively manages pore growth, forming a favorable structure dominated by uniformly distributed closed pores. When the multiplier exceeds 100%, the control over the structure diminishes, leading to deteriorated homogeneity and an increased proportion of open pores. This results in diminishing returns for mechanical strength, durability, and thermal insulation efficiency. Therefore, from a microstructural optimization perspective, a foaming multiplier of 100% represents the critical threshold for achieving a balanced pore structure within the studied system.

Based on Figure 10 and Table 6, there is a significant positive correlation between the foaming factor and the average pore diameter. As the foam content increases, the uniformity of the pore structure gradually deteriorates. The main pore diameter distribution range significantly expands, from 10–80 μm (F0) to 100–800 μm (F150). This large variability implies a high degree of heterogeneity in the pore groups. The observations for samples F100 and F150 directly attribute this loss of homogeneity to the emergence of large pores and significant pore merging, consistent with the instability and coalescence phenomena of foams under high gas volumes.

This transition from a dense and uniform microscopic structure (F0, F25) to a rougher and less uniform microscopic structure (F100, F150) provides a direct microscopic structural explanation for the macroscopic performance trend. The formation of larger pores and the expansion of the size distribution reduce the proportion of the load-bearing solid fraction and generate stress concentration points, directly leading to the observed decrease in compressive strength. Meanwhile, although an increase in porosity usually reduces thermal conductivity, in the case of a high foaming factor (F150), excessive pore merging and increased open porosity may reduce the insulation efficiency per unit density decrease. Therefore, these data indicate that a 100% foaming factor may represent a critical balance point, achieving a significant increase in average pore size for insulation purposes while still maintaining a certain degree of structural integrity, which is lost at 150%.

Future research will employ automated image analysis software (e.g., ImageJ 1.54g) to obtain precise quantitative data on porosity, pore size distribution, and pore circularity, enabling more rigorous structure–property modeling.

## 4. Summary

(1) The incorporation of highly stable modified foam significantly reduces the density of ultra-lightweight magnesium oxide-based foam concrete. The density of the magnesium oxide foam concrete can be effectively regulated by adjusting the proportion of the foam stabilizer xanthan gum (XG) to the magnesium oxide foaming agent (GX-7) and the total foam dosage. When a foam stabilizer-to-foaming agent ratio of 0.5:0.5:100 was used and the foam content reached 100% (relative to the mass of magnesium oxide powder), the dry density of the ultra-lightweight magnesium sulfate-based foam concrete decreased to 136.3 kg/m^3^, representing a reduction of 95.55% compared to the non-foamed reference.

(2) The compressive strength of ultra-lightweight magnesium oxysulfate foam concrete exhibited a strong inverse correlation with its total porosity. As the foam content increased, the overall porosity rose substantially, which directly reduced the load-bearing solid fraction per unit volume and consequently lowered the compressive strength. Furthermore, excessive foam content (foaming multiplier > 100%) led to increased pore coalescence and rupture, degrading the uniformity and structural integrity of the pore network. From a microstructural perspective, the increase in porosity not only diluted the volume fraction of the cementitious matrix but also physically confined the space available for the development of hydration products. This restriction significantly inhibited the formation of the primary strength-contributing crystalline phase, 5Mg(OH)_2_·MgSO_4_·7H_2_O (5·1·7 phase), further accentuating the decline in mechanical performance. Within the studied range, a foam content of 100% yielded an optimal pore structure that balanced a high porosity (for low thermal conductivity) with sufficient microstructural integrity to retain mechanical strength suitable for non-load-bearing insulation applications.

(3) At a foam content of 125%, the ultra-lightweight magnesium oxysulfate foam concrete exhibited a thermal conductivity of 0.081 W/(m·K), corresponding to a 96.36% reduction compared to the non-foamed reference material.

(4) Owing to its comparatively low mechanical strength, the material in its current state is not suitable for immediate practical application. This performance limit, however, serves as the basis for subsequent research, in which the incorporation of straw frameworks has been explored and has demonstrated a measurable enhancement in mechanical properties. Additionally, future work will prioritize enhancing the water resistance of MOS foam concrete, employing strategies such as the incorporation of hydrophobic admixtures, the application of surface coatings, or chemical modification of the MOS matrix.

## Figures and Tables

**Table 1:** Mixing proportions of high-stability foams (by mass relative to MgO).

	XG (g)	GX-7 (g)	H_2_0 (mL)
1-1	0.3	0.3	100
1-2	0.3	0.5	100
1-3	0.3	0.7	100
2-1	0.5	0.3	100
2-2	0.5	0.5	100
2-3	0.5	0.7	100
3-1	0.7	0.3	100
3-2	0.7	0.5	100
3-3	0.7	0.7	100

Note: “XG” stands for xanthan gum. “GX-7” represents magnesite foaming agent. “CA” represents citric acid. “1-” indicates the same mass of xanthan gum for the base mixture. For different batches of GX-7, this pattern is followed accordingly.

**Table 2:** Mixing proportions of ultralightweight magnesium sulfate foamed concrete (by mass relative to MgO).

	MgO (g)	MgSO_4_·7H_2_O (g)	H_2_O (mL)	CA (g)	Foaming Agent (g)
F0	135	60	60	1.35	0
F25	135	60	60	1.35	33.75
F50	135	60	60	1.35	67.5
F75	135	60	60	1.35	101.25
F100	135	60	60	1.35	135
F125	135	60	60	1.35	168.75

Note: “F25” indicates that the added foaming agent constitutes 25% of the MgO mass. “F50” indicates that the added foaming agent constitutes 50% of the MgO mass. And so on.

**Table 3:** 5-h and 9-h bleeding rates.

	XG (g)	GX-7 (g)	H_2_0 (mL)	5 h Bleeding Rate	9 h Bleeding Rate
1-1	0.3	0.3	100	17%	67%
1-2	0.3	0.5	100	19%	80%
1-3	0.3	0.7	100	34%	95%
2-1	0.5	0.3	100	11%	73%
2-2	0.5	0.5	100	4%	44%
2-3	0.5	0.7	100	9%	48%
3-1	0.7	0.3	100	28%	91%
3-2	0.7	0.5	100	19%	71%
3-3	0.7	0.7	100	21%	73%

Note: “XG” stands for xanthan gum. “GX-7” represents magnesite foaming agent. “CA” represents citric acid. “1-” indicates the same mass of xanthan gum for the base mixture. For different batches of GX-7, this pattern is followed accordingly.

**Table 4:** Dry density for each series.

Group	F0	F25	F50	F75	F100	F125
Dry density (kg/m^3^)	1847	1278	629	306	248	137

**Table 5:** The thermal conductivity of common insulation materials available on the market.

Name of the Material	Aerated Concrete	Straw Pulp Board	The Research Materials	Foam Glass	Mineral Wool	Rock Wool
Thermal conductivity/(W/(m·K))	0.098~0.12	0.068~0.084	0.081	0.066	0.053	0.036~0.041

**Table 6:** Statistics of Porosity Parameters.

Number of Samples	Average Aperture (μm)	Main Range of Aperture Distribution (μm)	Remarks
F0	<50	10–80	Dense structure, with gel pores as the main component
F25	50 ± 25	50–150	The distribution is concentrated and the pore structure is uniform.
F50	80 ± 40	100–300	As the aperture increases, the distribution begins to broaden.
F100	200 ± 150	150–500	A few large pores appeared, and the uniformity decreased.
F150	420 ± 250	100–800	Extremely wide distribution, with a significant phenomenon of pore merging

## Data Availability

The original contributions presented in this study are included in the article. Further inquiries can be directed to the corresponding author.
